# Free space super focusing using all dielectric hyperbolic metamaterial

**DOI:** 10.1038/s41598-020-61639-2

**Published:** 2020-07-13

**Authors:** Norhan A. Salama, Mai Desouky, S. S. A. Obayya, Mohamed A. Swillam

**Affiliations:** 1 Laser Application in Metrology, Photochemistry & Agriculture, National Institute of Laser Enhanced Sciences, Cairo University, Giza, Egypt; 2 Department of Physics, The American University in Cairo, Cairo, 11835 Egypt; 3 Centre for Photonics and Smart Materials, Zewail City of Science and Technology, Giza, Egypt

**Keywords:** Nanoscience and technology, Nanoscale materials, Metamaterials

## Abstract

Despite that Hyperbolic Metamaterial (HMM) has demonstrated sub-wavelength focusing inside of it, sub-wavelength imaging in free space of HMM is rarely introduced. The decay of hyperbolic momentum space outside the hyperbolic medium has hindered the realization of sub-wavelengh focusing in the near field of HMM. Furthermore, manipulating the negatively refracted waves exiting the HMM have addressed another major obstacle to realize free space sub-wavelength focusing. In this work, we report extended sub-wavelength focusing in free space based on negative refraction of light exiting the HMM. The proposed structure is composed of multilayers of doped InAs/intrinsic InAs integrated with metallic slit. We theoretically simulate the doped InAs/intrinsic InAs HMM and investigate the negative refraction behavior outside the HMM. We optimized the structure for achieving high resolution down to 0.2λ, extended to a distance of 3.2 µm in free space. Also, sub-wavelength focusing in free space has been studied at different doping concentrations showing that the small doping concentrations exhibit enhancement in resolution at short distances up to 600 nm away from the HMM. Extending the focusing distance is achieved up to distance 3.5 µm from the hyperbolic structure by manipulating the doping concentration. This proposed lens configuration is expected to find potential usage in mid IR thermal imaging and photolithography application.

## Introduction

Imaging with conventional optical systems is constrained with the diffraction limit, where the maximum resolution can be reached is at most half of the incident wavelength. Imaging is based on retrieving the scattering amplitudes and phase shifts of various momentum modes. In ordinary isotropic medium, high orders of momentum components (high *k*-modes) -which are larger than those of free space (K_o_)-exponentially decay in the vicinity of the object, leading to exponential small light interaction with the object. Consequently, the scattering from these modes are negligible and evanescent in free space, thus, they can’t contribute to the formation of the final image of an object. These high *k*-modes carry sub-wavelength features of the object^1^. In order to beat the diffraction limit, it is required to recover the high order momentum modes that are exponentially decayed in free space, thus, retrieving the sub-wavelength features of an object. Many studies have introduced different approaches to overcome the diffraction limit such as the contact mask and scanning near field optical microscopy (SNOM)^2,3^. However, they all suffer from the drawbacks of low throughput and long post processing. Alternatively, metamaterial, as first proposed by Veselago (1968)^4^ represents a new paradigm striking the electromagnetic features in which it exhibits negative refraction of light, a property that cannot be attained before. The super lens, proposed by sir.Pendry^5^, was the first demonstration of Veslago’s theory enabling sub-diffraction resolution imaging in microwave wavelength range. Although, super lens is a successful approach overcoming the diffraction limit, serious constrains are imposed by nanofabrication techniques, when uplifting the working frequencies of super lens up to optical ranges^6^. That is in addition to the difficulty to obtain negative magnetic permeability in the visible domain^7^. Inspired by Pendrys’ work, different studies have developed different approaches for tailoring the electric permittivity and magnetic permeability and hence the index of refraction. These approaches find multiple functionalities such as invisibility cloacking^8^, perfect absorption^9^, digital coding^10–12^, negative refraction^13^ sub-diffraction focusing^14–18^. Hyperbolic metamaterials (HMMs) can act as an alternative solution to achieve sub-diffraction focusing without the need to tune the magnetic permeability or implementing complex designs. HMM is a composite structure that displays a dispersion relation where the material behaves as a metal in one direction in space and as a dielectric in the other direction^19,20^, such material is also called indefinite media. Hyperlens, a curved structure that can be used for sub-diffraction imaging in the far field^21^. However, the difficulty of fabricating the curved hyperlens restricts its practical application. Despite of the demonstration of numerous studies concerning sub-diffraction focusing inside the HMM medium^22–26^, lensing in the near field of HMMs is rarely introduced in real structures except for few attempts - such as Si pillars arrays in the THz range^27^- providing minor focusing resolution. The challenge of near field focusing outside the HMM is imposed by the rapid decay of the evanescent waves in free space. Nonetheless, HMM is characterized by extremely large optical density of states that extended to the near field of HMM and consequently do contribute to the propagation of large wave vectors outside the HMM that is in turn, prevents the rapid decay of the evanescent field^28^. This phenomenon is accompanied with subsequent negative refraction of the electromagnetic (**EM**) waves exiting the HMM, which are the key roles for achieving sub diffraction focusing in the near field of HMM^6^. In this work, we report near field focusing in free space with sub-diffraction focusing resolution based on a single planar HMM lens composed of doped InAs/intrinsic InAs integrated with upper metallic slit. The proposed design can be fabricated using conventional 2D- fabrication technologies such as e-beam lithography and evaporation of metal films, or using direct Laser writing^29–31^, the procedure has been explicitly described in previous reference^32^.

We demonstrate the sub-diffraction focusing effect based on paraxial negative refraction (small angle of refraction) across the second interface of the HMM lens for structure of doping concentration (N_d_) 7.5 × 10^19^ cm^−3^. Sub-diffraction focusing of resolution down to 0.16λ at λ = 7.6 µm has been achieved at focusing plane lies 500 nm away from the HMM lens. In addition, we report tuning the focusing distance away from the HMM lens through optimizing the slit parameters with maximum distance reached is 3.2 µm at wavelength of 7.6 µm and resolution down to 0.2λ. Furthermore, we investigate the focusing effect of different doping concentrations showing that the maximum extended projection for focusing is 3.5 µm achieved for N_d_ of 7.5 × 10^19^ cm^−3^ at 17.74 µm.

## Theoretical Design

**Figure 1:**
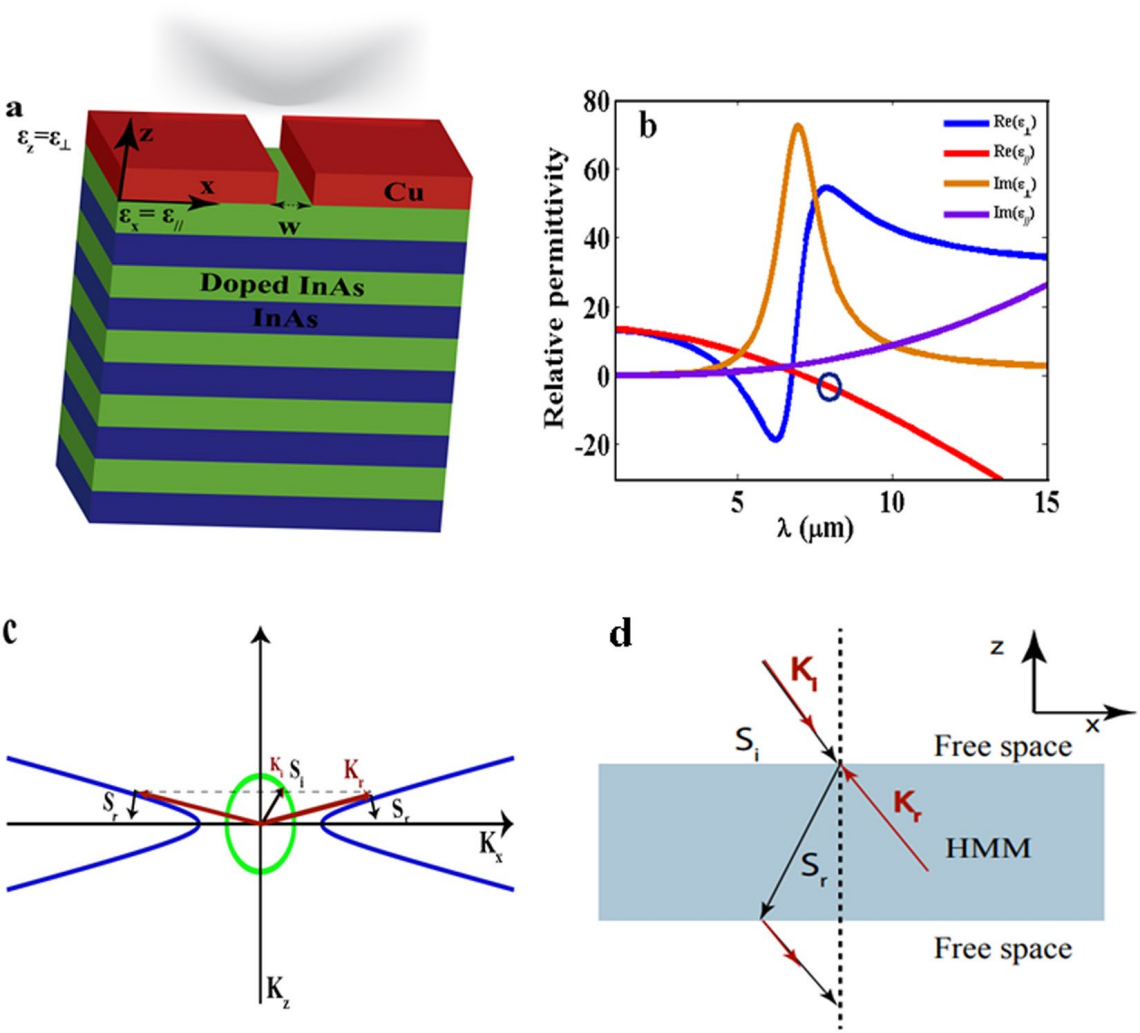
A schematic of the proposed HMM. It consists of 10 alternating layers of doped InAs/intrinsic InAs, with layer thickness of 20 nm each. A Cu layer integrated with a slit on top of the structure. The effective permittivities were defined such that ${{\boldsymbol{\varepsilon }}}_{//}$ is along the x-axis and ${{\boldsymbol{\varepsilon }}}_{\perp }$ is along the z-axis. (**b**) The dispersion relation of doped InAs/intrinsic InAs of N_d_ of 7.5 × 10^19^ cm^−3^, the blue circle indicates the wavelength region of interest verifying type (П) HMM, where the real part of in-plane effective permittivity is $\,{{\boldsymbol{\varepsilon }}}_{//} < 0\,$(red curve), while the real part of out-of-plane effective permittivity is ${{\boldsymbol{\varepsilon }}}_{\perp } > 0\,$(blue curve). (**c**) The open hyperboloid equi-frequency contour at λ** =** 7.6 µm with $\,{{\boldsymbol{\varepsilon }}}_{//}$ = −**1.889** and ${{\boldsymbol{\varepsilon }}}_{\perp }=52.65\,$with corresponding Poynting vector which is orthogonal to the iso-frequency contour as well as the equi-frequency contour of an isotorpic material (green circle) and (**d**) A schematic represents the refraction behaviour throughout the HMM lens based on the direction of Poynting vectors (S) and wave vector (K).The incident wave vector is given by K_i_ and the incident Poynting vector is given by S_i_. The first refraction takes place inside the HMM when the Poynting vector (S_r_) is negatively refracted with respect to x-axis while the wave vector will be propagating backward. After exiting the HMM, the S_r_ will undergo secondary negative refraction leads to beam focusing while being parallel with the K_r_.

The structure is composed of 10 alternating layers of doped/intrinsic InAs layers of 20 nm each, integrated with upper metallic slit (copper slit), see Fig. 1a. In order to excite the high wave vectors in a HMM, a diffraction grating or Fresnel zone plate is applied on top of the structure. The upper slit ***w*** is tuned for optimum focusing. We define the permittivity tensor components as diagonal tensor with (ɛ_xx_,ɛ_yy_,ɛ_zz_) corresponds to (ɛ_//_,ɛ_//_,ɛ_⊥_). The parallel permittivity (ɛ_//_) corresponds to the in-plane component and the perpendicular permittivity (ɛ_⊥_) corresponds to the out of plane component. The commercial software finite difference time domain-Lumerical- is used for theoretical investigation^33^. TM polarized Gaussian beam of NA = 0.2 is incident from the top of the structure providing paraxial incidence (small angle of incidence). Perfect matched layers (PML) are used as the boundaries in the x and z directions. The structure has sub-wavelength dimensions, so, its effective parameters could be extracted by homogenization using the effective medium approximation (EMA) theory as follows^34^:

1 $${\varepsilon }_{\perp }=\frac{{\varepsilon }_{doped}\cdot {\varepsilon }_{d}}{{f}_{1}{\varepsilon }_{doped}+{f}_{2}{\varepsilon }_{d}}$$

2 $${\varepsilon }_{\Vert }={f}_{1}{\varepsilon }_{doped}+{f}_{2}{\varepsilon }_{d}$$

where *ɛ*_*doped*_ and *ɛ*_*d*_ are the permittivities of doped and intrinsic InAs respectively. The filling ratios *f*_1_ and *f*_2_ are chosen to be 0.5. The permittivity of intrinsic InAs is ɛ_d_ = 3.937, while the permittivity of doped InAs is calculated using the Drude modal.

3 $${\varepsilon }_{doped}={\varepsilon }_{\infty }-\frac{{\omega }_{p}^{2}}{{\omega }^{2}+i{\omega }^{2}\Gamma }$$

where *ɛ*_*∞*_ = 15.5, *ω*_*p*_ is the plasma frequency and *Γ* is the scattering rate. The values of *ω*_*p*_ and *Γ* are extracted from previous literatures^27,35^. The dispersion relation of the structure comprising InAs of doping concentration N_d_ 7.5 × 10^19^ cm^−3^ is presented in Fig. 1b. The hyperbolic region of type (II) where (ɛ_//_ < 0 and ɛ_⊥_ > 0) is the region of interest throughout our whole study (marked by blue circle in Fig. 1b).

The key for near field sub-diffraction focusing using HMM is based on two aspects: the first aspect is, the extreme anisotropic nature of HMM, i.e. where *ɛ*_*zz*_*.ɛ*_*xx*_ < *0*, that enables the sustained propagation of high *k*-modes inside the HMM and could be extended to a distance in the near field (outside the HMM) comprising the high optical density of states. The extreme anisotropic nature of HMM gives rise to open hyperboloid iso-frequency contour, as demonstrated in Fig. 1c, according to the dispersion relation:

4 $$\frac{{k}_{x}{}^{2}+{{k}_{y}}^{2}\,}{{\varepsilon }_{zz}}+\frac{{{k}_{z}}^{2}\,}{{\varepsilon }_{xx}}=\frac{{\omega }^{2}}{{c}^{2}}$$

The second aspect is based on the negative refraction behaviour across the interface of HMM/free space^6,36,37^. For paraxial incidence (verified by Gaussian beam), the excited high *k*-modes preferentially propagates inside HMM with an angle of refraction, with respect to the optical axis, defined by the ratio of components of Poynting vectors, according to the following relation^38^:

5 $$\theta =\arctan \frac{{S}_{x}}{{S}_{z}}=\arctan \left(\frac{{\varepsilon }_{xx}{k}_{x}}{{\varepsilon }_{zz}kz}\right)$$

when ${k}_{x}\to \infty$, the limit of the angle is approximated as follows:

6 $${\theta }_{{\rm{l}}{\rm{i}}{\rm{m}}}=\arctan \left(\sqrt{\frac{{\varepsilon }_{xx}}{{\varepsilon }_{zz}}}\right)$$

From Eq. (6), we can conclude that the refraction across the first HMM lens interface is a function of uniaxial parameters of the lens irrespective to the incident angle^22^. The first refracted modes (K_r_) are propagating inside the HMM lens striking the second interface of the lens and then undergoing further refraction, Fig. 1d. The negative refraction behavior can be explained upon understanding the direction of Poynting vector components S_x_ (the tangential component of Poynting vector) and S_z_ (the component in the direction of propagation representing the energy flow) across the interface. The energy flow has to be away from the interface, thus, S_z_ > 0^39^. The real part (Re) of the two components of Poynting vectors are represented by the following inequality equations^38,40,41^:

7 $$\langle S\rangle =1/2{\rm{R}}{\rm{e}}\langle {S}_{x},0,{S}_{z}\rangle $$

where the $\hat{S}$ in the direction of energy flow is given by,

8 $${S}_{z}=\frac{{k}_{z}}{{\varepsilon }_{xx}}\frac{{{H}_{o}}^{2}}{2\omega {\varepsilon }_{o}}\succ 0$$

and the tangential component of $\widehat{S,}$

9 $${S}_{x}=\frac{{k}_{x}}{{\varepsilon }_{zz}}\frac{{{H}_{o}}^{2}}{2\omega {\varepsilon }_{o}}\prec 0$$

10 $$\langle S\rangle =\frac{{{H}_{o}}^{2}}{2\omega {\varepsilon }_{o}}{\rm{R}}{\rm{e}}\langle \frac{{k}_{x}}{{\varepsilon }_{zz}},0,\frac{{k}_{z}}{{\varepsilon }_{xx}}\rangle $$

From (10), Eq. (5) can be rewritten as follows:

11 $$\tan \theta =\frac{{S}_{x}}{{S}_{z}}=\left(\frac{{k}_{x}/{\varepsilon }_{zz}}{{k}_{z}/{\varepsilon }_{xx}}\right)$$

In our study, we use a HMM where (ɛ_xx_ < 0 and ɛ_zz_ > 0), thus from Eq. (11), *S*_*z*_ has to be positive and the sign of *S*_*x*_ flips across the interface and the energy is negatively refracted between the HMM and free space. The wave vector components become *k*_x_ < 0 and *k*_z_ < 0, thus, the wave front which is represented by the wave vector, is negatively refracted with respect to x-component and also, undergoes backward propagation according to the negative z-component. In analogy to the super lens, as proposed by Sir.Pendry, HMM can possibly allow for secondary sub-wavelength focusing in free space based negative refraction. When the EM exits the lens in the positive dielectric medium, it is negatively refracted with respect to the wave inside the lens, thus leading to secondary focusing in the near field of the HMM. Figure 1d demonstrates the travelling wave vector and Poynting vector of a single ray throughout the HMM.

## Results and Discussion

**Figure 2:**
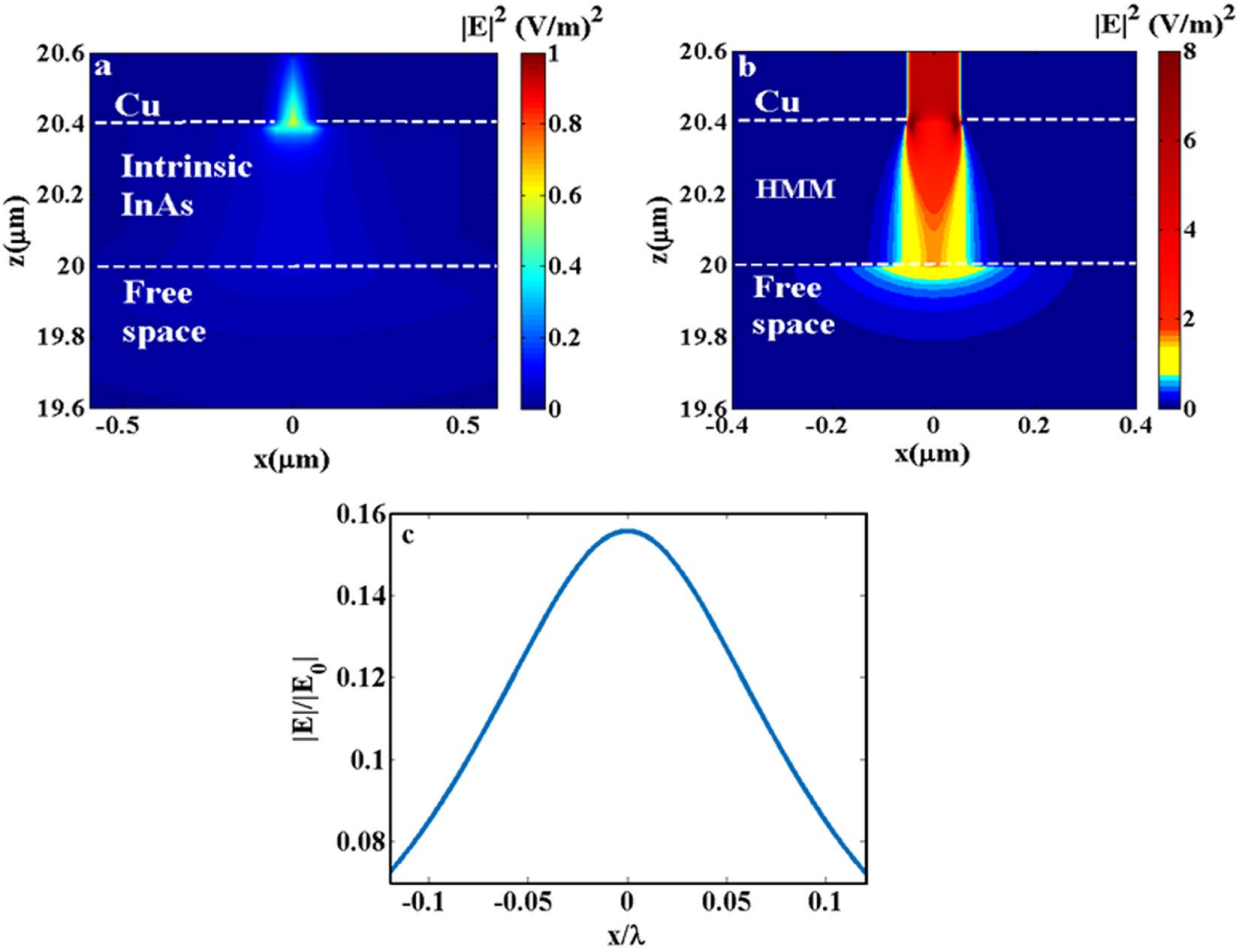
The electric field intensity distributions in xz-plane for: (**a**) Intrinsic InAs and (**b**) effective HMM structure, both are at λ = 7.09 µm with Cu slit on top of both structures. The field can’t propagate inside the intrinsic InAs while it propagates and get focused inside and outside the HMM. (**c**) The electric field intensity profile at a focal plane 500 nm away from the HMM, indicating sub-diffraction focusing of the transmitted field in free space.

We verify the effect of focusing in the near field of HMM through a comparison of the electric field distribution throughout two structures: intrinsic InAs and HMM lens using effective medium approximation theory, Fig. 2. It is clearly observed that there is almost no propagation of the electric field in intrinsic InAs while, in HMM, the electric field travels throughout and transmits out of HMM in form of focused waves. Figure 2c confirms the focusing effect through the normalized electric field profile measured at a plane lies 500 nm away from the HMM.

**Figure 3:**
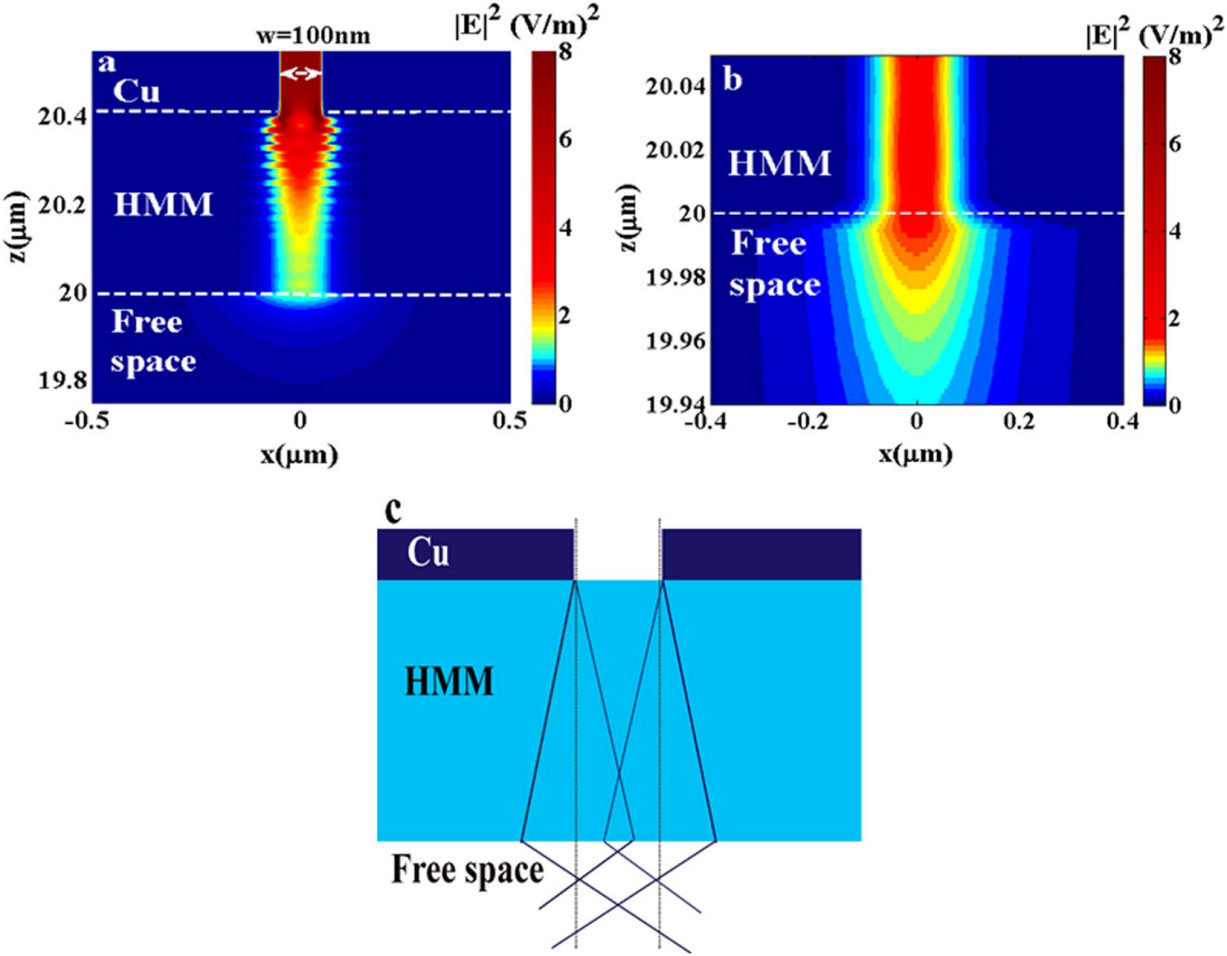
The electric field intensity distributions in xz-plane: (**a**) the whole structure is of slit width 100 nm and (**b**) zoom in across the HMM/free space interface, confirms that the propagating field undergoes negative refraction in the near field of the HMM lens and (**c**) The schematic is presenting the ray tracing diagram throughout the structure; the dashed lines represent the optical axes, the diagram shows the expected negative refraction in the near field of the HMM.

In the next section, we apply the real structure and investigate the sub-diffraction focusing effect in the near field, Fig. 3. Figure 3(a,b) shows that the major part of the electric field which is excited at both edges of metallic slit, travels throughout the HMM with a small angle of refraction with focusing inside the lens and also extended outside the lens. Part of the electric field transmits outside the lens and gets focused in the near field. Figure 3b represents zoom in across the HMM/free space interface confirming the negative refraction behavior outside the lens, and thus focusing in the near field of the lens. It is observed in the same figure the presence of broadening in the electric field in the very near field of the lens. This broadening is confined within very short distance, so called transition distance. The transition distance is the distance at which the EM behaviour is in between the coupled surface plasmon polariton in multilayered structure –which can sustain the propagation of the high wave vectors and results in bulk surface plasmon polariton (BSPP) -, and free space where the high *k*-modes start to exhibit different dielectric permittivity (positive medium). The BSPP provides enhanced localized density of states (LDOS) in the very near field of the structure^28^. As a result, the homogenization of the HMM within the transition distance breaks down and the propagating large wave vectors do contribute with deviation to the emerged large LDOS. After the transition distance, the HMM exhibits homogenization model and the propagating wave vectors start to propagate with negatively refraction behavior verifying a secondary focusing point in the near field. In ref. ^28^, the transition distance is estimated to be around a/2pi –where a is the layer thickness- which is in good agreement with our results where the transition distance in the proposed structure is around 3 nm, Fig. (3b).

Figure 3c shows a schematic for the ray tracing explaining the secondary negative refraction in the near field of HMM.

**Figure 4:**
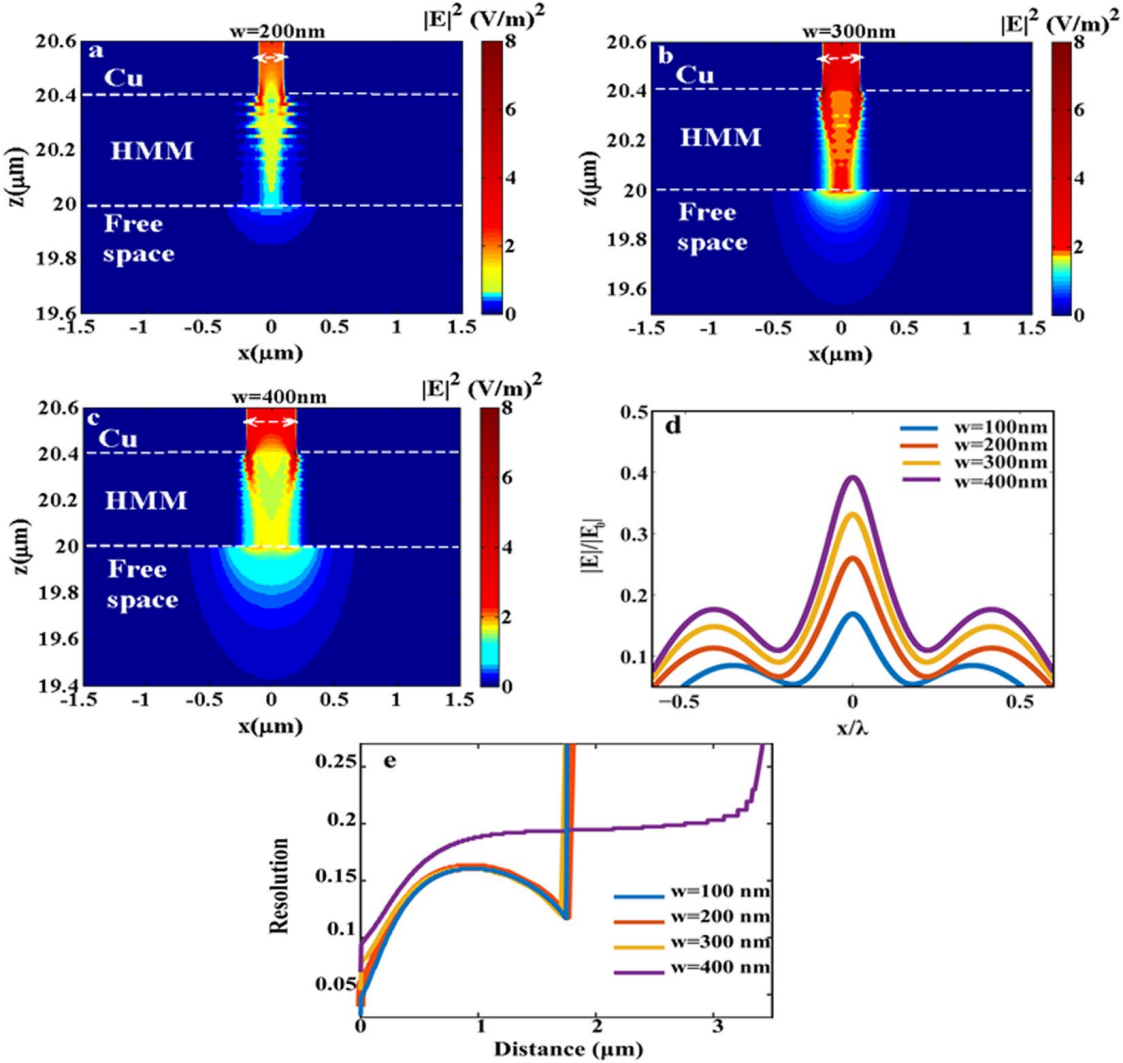
The electric field intensity distributions in xz-plane throughout a range of *w*: (**a**) 200 nm, (**b**) 300 nm, (**c**) 400 nm, demonstrating the focusing effect. (**d**) The normalized electric field profile in a plane at distance 500 nm below the HMM lens showing the direct relation between the ***w*** and amplitude of the normalized electric field while the focusing resolution is almost fixed around (0.15λ – 0.16λ) and (**e**) The focusing resolution against the distance away from the HMM lens showing the superior extended focusing of HMM of ***w*** of 400 nm to a distance of 3.2 µm, despite of its least resolution than other structures in short distances.

Now, we investigate the focusing resolution and the focusing resolution distance away from the HMM lens. We optimize the slit width ***w*** for obtaining the best focusing resolution at a plane lies 500 nm away from the HMM lens and also obtaining the maximum focusing distance that is possible away from the HMM lens. The focusing resolution is determined by the full width at half maximum (FWHM) of the electric field profile with respect to the incident wavelength (x/λ).The focusing effect is achieved within a range of ***w*** between 100 nm to 400 nm, Fig. 4. Figure 4(a–c) shows that the focusing resolution almost doesn’t change with changing the ***w*** from 100 to 300 nm with focusing resolution down to 0.15λ, except for ***w*** of 400 nm, where the resolution slightly changes to 0.16λ. However, the amplitude of the electric field is increased with increasing the slit width, Fig. 4d. Moreover, the structure of ***w*** 400 nm exhibits extended focusing distance to 3.2 µm, Fig. 4e. Figure 4e also shows that the resolution for the structures of ***w*** 100–300 nm decreases along the focal axis up to a point. After this point, the resolution starts to increase and then vanishes. This can be explained upon the demonstration of the scheme of the ray tracing, Fig. 3c. The scheme shows the arising of two separate focusing points. The first focusing point is arisen from the negative refraction of the focused rays inside the HMM. Meanwhile, the second focusing point is arisen from the negative refraction of the unfocused rays inside HMM. The region between the two focusing points exhibiting the least resolution as it is an extension of the two focusing points. For the structure of ***w*** 400 nm, it seems that the second focusing point is not well formed as the previous cases. Instead, the rays travel with negative refraction for long distance (3.2 µm) exhibiting small resolution. Thus, there is a direct relation between the slit width and the focusing condition to be achieved. The slit width needs to be designed in away such that the first refracted rays with slight angles of propagation can interfere in a point inside the HMM in order to form the first focusing point outside the HMM. Also, the first refracted unfocused rays with slight angles of propagation inside the HMM can be narrow enough to project a second focusing point in the near field of the HMM. Then the refracted rays exiting the HMM undergoes secondary focusing, based on an opposing refraction to the rays inside the HMM. Thus, optimizing the ***w*** is a crucial parameter for the near field focusing outside the HMM.

**Figure 5:**
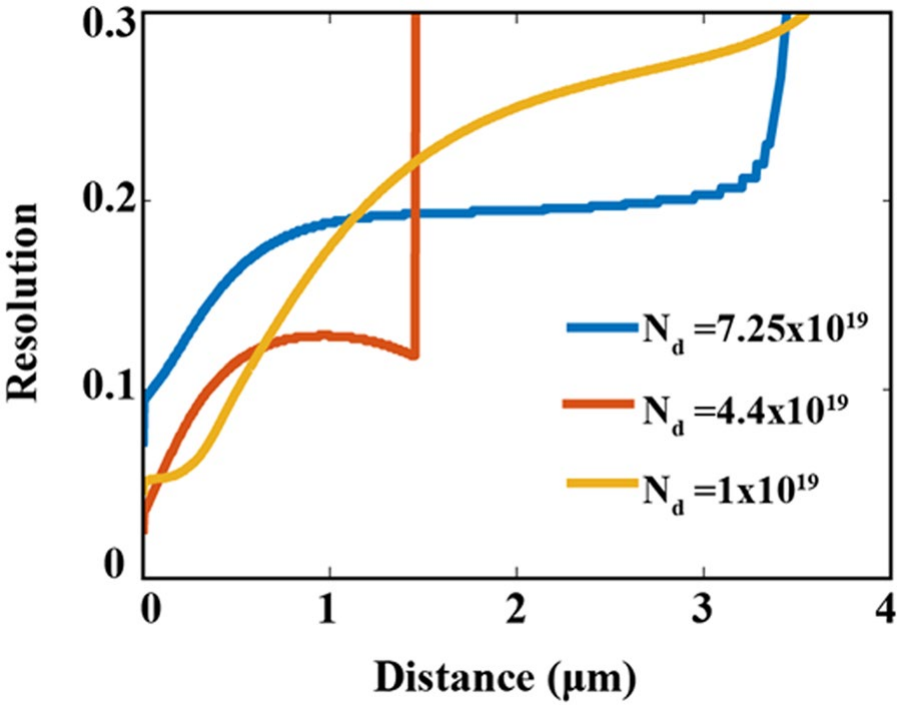
The focusing resolution against the distance away from HMM lens of slit width *w* of 400 nm for different doping concentrations (N_d_) of 7.25 × 10^19^, 4.4 × 10^19^, 1 × 10^19^ cm^−3^ corresponding to focusing wavelengths of 7.6 µm, 9 µm and 17.74 µm, respectively.

Finally, we examine focusing resolution with respect to the distance away from the HMM using different doping concentrations (N_d_) of 7.5 × 10^19^ cm^−3^, 4.4 × 10^19^ cm^−3^ and 1 × 10^19^ cm^−3^ corresponding to wavelengths of 7.6 µm, 9 µm, and 17.74 µm respectively, (these wavelengths have been selected based upon the best focusing resolutions achieved). Figure 5 and Table 1 show that the maximum sub-diffraction focusing distance achieved for each doping concentration is: 3.2 µm, 1.45 µm and 3.5 µm, respectively. At short distances up to 600 nm, the region of the first elongated focusing point, the focusing resolution is greatly enhanced by decreasing the doping concentration. The reason behind is that by decreasing the doping concentration, the metallic property of the structure decreases and hence the imaginary part of the index of refraction that defines the losses decreases. However, at large distances beyond 600 nm, the resolution is less affected by the metallic property and losses. The different doping concentrations exhibit different behaviour along the focal axis. In case of (N_d_) of 4.4 × 10^19^ cm^−3^, the focusing resolution is enhanced at large distances due to the formation of the secondary focusing point as previously explained. In contrary, the case of (N_d_) of 7.5 × 10^19^ cm^−3^ and 1 × 10^19^ cm^−3^, the focusing resolution gradually decreases till vanishes. Thus, the control of energy losses emerged from either doping concentration or even by fabrication roughness is crucial for enhancing the focusing resolution in the near region of the near field of HMM.

**Table 1:** Summary of focusing effects according to the inside angle of propagation, the focusing resolution at a plane lies 500 nm away from the HMM surface, and the maximum focusing distance with respect to different doping concentration at their best focusing wavelengths.

N_d_(cm^−3^)	Focusing wavelength (μm)	Theoretical inside angle of refraction (θ)	Focusing resolution at (500 nm)	Maximum focusing distance (μm)
7.25 × 10^19^	7.6	10.9°	0.19λ	3.2
4.4 × 10^19^	9	3.79°	0.11λ	1.45
1 × 10^19^	17.74	7.03°	0.099λ	3.5

Table 1 summarizes the values of the inside angle of refraction, the focusing resolution at a plane lies 500 nm away from the HMM, and the maximum extended focusing distance with respect to different doping concentration with their best focusing wavelengths indicated.

## Conclusion

In this work, we theoretically demonstrate near field sub-diffraction focusing in free space using HMM lens based on InAs semiconductors in the mid-IR range. Focusing is achieved using HMM type (П) as they support paraxial propagation of high *k*-modes. Hence, negative refraction of energy is achievable across the interface of HMM\free space and consequently sub-diffraction focusing in the near field is realized. We optimized the structure parameters for achieving the highest resolution and maximum focusing distance away from the HMM lens with best achieved resolution down to 0.2λ at maximum distance of 3.2 µm for N_d_ of 7.25 × 10^19^ cm^−3^. Upon using different doping concentrations, we find that the small doping concentrations enhance the resolution in short distances from the HMM up to 600 nm. The sub-diffraction resolution for N_d_ of 7.25 × 10^19^ cm^−3^, 4.4 × 10^19^ cm^−3^, 1 × 10^19^ cm^−3^ at wavelengths of 7.6 µm, 9 µm and 17.74 µm are 0.16λ, 0.11λ, and 0.099λ respectively projecting maximum focusing distances up to 3.2 µm, 1.45 µm and 3.5 µm, respectively. Our study engenders new paradigms to realize mid-IR metalens for thermal imaging and photolithography application.

## Materials and Methods

Finite difference time domain (lumerical software) has been used for simulating the optical response of the proposed structures to incident plane waves. Material modeling using Drude model has been performed using Matlab.
